# Characterization of a REST-Regulated Internal Promoter in the Schizophrenia Genome-Wide Associated Gene MIR137

**DOI:** 10.1093/schbul/sbu117

**Published:** 2014-08-25

**Authors:** Alix Warburton, Gerome Breen, Dan Rujescu, Vivien J. Bubb, John P. Quinn

**Affiliations:** ^1^Department of Molecular and Clinical Pharmacology, Institute of Translational Medicine, University of Liverpool, Liverpool L69 3BX, UK;; ^2^King’s College London, MRC Social Genetic and Developmental Psychiatry Research Centre, Institute of Psychiatry, London, UK;; ^3^National Institute for Health Research (NIHR), Biomedical Research Centre for Mental Health, South London and Maudsley NHS Foundation Trust and Institute of Psychiatry, King’s College London SE5 8DF, UK;; ^4^Department of Psychiatry, University of Halle-Wittenberg, Halle, Germany

**Keywords:** cocaine, gene environment interaction, microRNA-137, variable number tandem repeat

## Abstract

MIR137 has been identified as a candidate gene for schizophrenia from genome-wide association studies via association with an intronic single nucleotide polymorphism (SNP), rs1625579. The location of the SNP suggests one mechanism in which transcriptional or posttranscriptional regulation of miR-137 expression could underlie schizophrenia. We identified and validated a novel promoter of the MIR137 gene adjacent to miR-137 itself which can direct the expression of distinct mRNA isoforms encoding miR-137. Analysis of both endogenous gene expression and reporter gene assays determined that this internal promoter is regulated by repressor element-1 silencing transcription factor (REST), which has previously been associated with pathways linked to schizophrenia. Distinct isoforms of REST mediate differential expression at this locus, suggesting the relative levels of these isoforms are important for miR-137 expression profiles. The internal promoter contains a variable number tandem repeat (VNTR) domain adjacent to the pre-miR-137 sequence. The reporter gene activity directed by this promoter was modified by the genotype of the VNTR. Differential expression was also observed in response to cocaine, which is known to regulate the REST pathway in SH-SY5Y cells. Our data support the hypothesis that a “gene × environment” interaction could modify the level of miR-137 expression via this internal promoter and that the genotype of the VNTR could modulate transcriptional responses. We demonstrate that this promoter region is not in disequilibrium with rs1625579 and therefore would supply a distinct pathway to potentially alter miR-137 levels in response to environmental cues.

## Introduction

Genome-wide association study (GWAS) for schizophrenia identified the intronic single nucleotide polymorphism (SNP) rs1625579 within the MIR137 gene to be strongly associated.^1^ The risk genotype for this variant is a strong predictor for earlier age-at-onset of psychosis and is associated with structural changes within the brain relative to carriers of the protective allele and healthy matched controls.^2^ MIR137 encodes for the microRNA-137 (miR-137), which functions in neurodevelopment, adult neurogenesis^3,4^ and is a validated regulator of GWAS candidate genes for schizophrenia,^1,5,6^ suggesting that impairments in the regulation and/or function of miR-137 may be a key mechanism in neuropsychiatric disease.

**Fig. 1.**
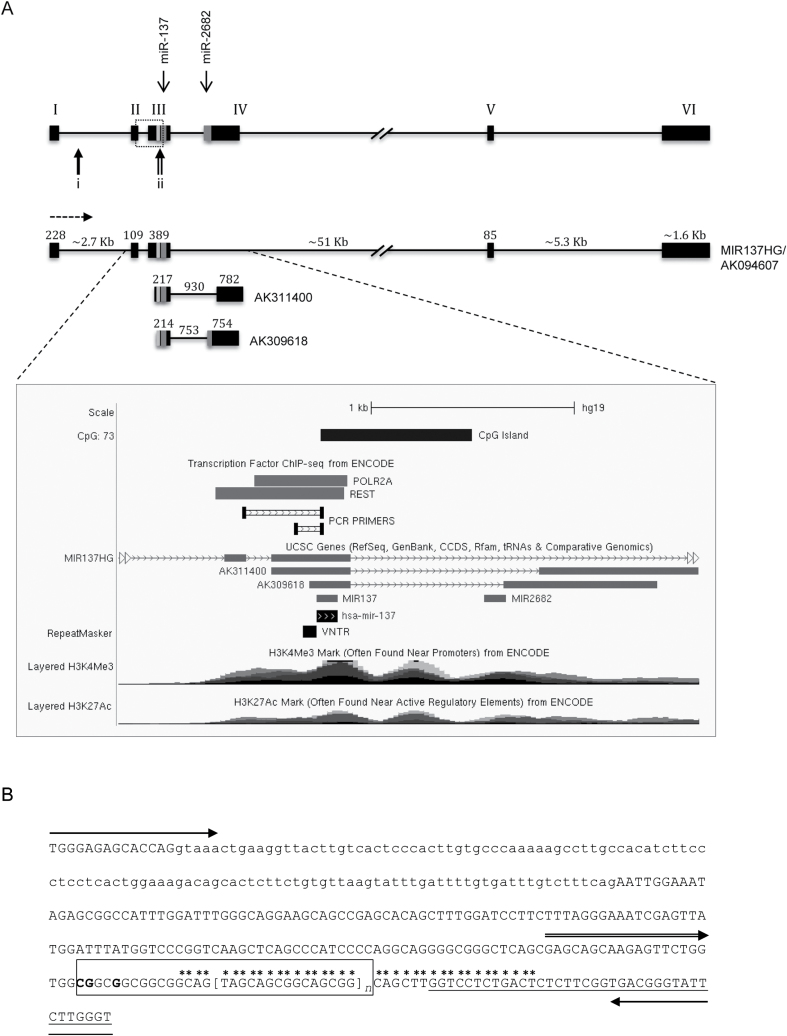
(A) Schematic showing the nonprotein coding genes MIR137HG (AK094607), AK311400, and AK309618 as described in the UCSC Genome Browser, Assembly GRCh37/hg19. Exons represented as black boxes; introns as connecting lines. The numbers above indicate size in bp, unless stated otherwise. The direction of transcription is indicated by a dashed arrow. MicroRNA (miR)-137 and miR-2682 are denoted by dark grey boxes. A 15-bp variable number tandem repeat (VNTR) immediately upstream of miR-137 is represented as a light grey bar. REST binding sites identified from ENCODE ChIP-seq data are marked as vertical arrows *i* and *ii*. *Lower panel*, Transcription factor binding and histone marks over the internal MIR137 promoter from ENCODE data. (B) Sequence targeted by PCR primers for the internal MIR137 promoter. PCR primer sequences are marked by horizontal arrows; the double horizontal arrow marks the alternative forward primer targeting the VNTR alone. Upper case font indicates exons and lower case font introns. The VNTR sequence is boxed; the repetitive element is marked with brackets, *n* representing copy-number variation. Underlined text indicates miR-137 sequence. Sequence marked with asterisks’ represents predicted REST binding sites identified using rVista 2.0 (http://rvista.dcode.org/).

Bioinformatic analysis of the MIR137 locus predicted an internal promoter adjacent to the miR-137 sequence (figure 1). This promoter encompassed a variable number tandem repeat (VNTR), which has previously been shown to modulate the processing and function of miR-137.^7^ VNTRs at other loci can be clinical correlates of psychiatric dysfunction.^8,9^ We postulated there to be transcriptional properties associated with the MIR137 VNTR because we have demonstrated that VNTRs can act as transcriptional regulators in both a tissue-specific and stimulus-inducible manner.^10–18^ ENCODE (Encyclopedia of DNA Elements) ChIP-Seq data indicated that repressor element-1 silencing transcription factor (REST),^19^ also termed neuron restrictive silencing factor (NRSF),^20^ was binding at this internal promoter. REST predominantly functions as a transcriptional repressor of neuronal gene expression through binding the RE1 (repressor element-1) or NRSE (neuron restrictive silencing element).^19,20^ Modulation of REST is associated with neurological dysfunction, including schizophrenia,^21^ with genetic variants being implicated in cognitive function.^22^ REST has several isoforms, most noticeably a truncated isoform known as sNRSF in human cells and related to REST4 in rodent cells, which has been shown to have distinct functions from full-length REST and whose expression is often associated with cellular stress or disease progression.^23–29^

In this communication, we address the transcriptional mechanisms that may operate at the proposed internal promoter to modulate cellular miR-137 levels. The resulting changes in miR-137 levels could underpin a pathway modulating schizophrenia.

## Methods

### Plasmid Construction

MIR137 fragments were cloned into the pGL3 Luciferase reporter vector system as detailed in supplementary S1.1.

### Cell Culture

Human SH-SY5Y neuroblastoma cells were maintained as described previously.^30^ For drug challenge, 10 µM cocaine or vehicle alone (sterile water) were diluted in appropriate volumes of cell culture media and added to the cells for 1 hour. For luciferase assays, drug treatments were performed 4 hours posttransfection.

### Luciferase Reporter Gene Assays

SH-SY5Y cells were seeded in 24-well plates at 100 000 cells per well and transfected with 1 µg plasmid DNA and 10 ng pMLuc2 (Novagen) (internal control for transfection efficiency) using TurboFect (Thermo Scientific). Transfected cells were processed 48 hours posttransfection using the Dual-Luciferase Reporter Assay System (Promega). Fold changes in firefly luciferase activity (normalized to *renilla* luciferase activity) supported by the MIR137 domains over the pGL3 controls were calculated and significance determined using one-tailed *t* tests. Significance was scored as follows */#*P* < .05, **/##*P* < .01, ***/###*P* < .001. For each transfection, *n* = 4.

### REST Overexpression Assays

For luciferase reporter gene assays, SH-SY5Y cells were processed with the addition of 1 µg RE-EX1 plasmid,^29^ expressing full-length human REST or sNRSF in the pcDNA3.1 vector system. For gene expression profiling, SH-SY5Y cells were seeded in 6-well plates at 400 000 cells per well and transfected with either 4 µg of RE-EX1 or pcDNA3.1_sNRSF. pcDNA3.1 alone was used as a negative control. Cells were incubated for 48 hours before being processed.

### RNA Extraction and Gene Expression Profiling

Total RNA was extracted using Trizol Reagent (Invitrogen). RNA concentration was determined using a NanoDrop-8000 spectrophotometer and 2 µg reverse transcribed into cDNA using the GoScript RT system. The total reaction volume of 20 µl was diluted 20× in nuclease free water and 1 µl cDNA used per 25 µl PCR reaction using GoTaq Flexi DNA Polymerase and PCR primers for the MIR137 transcripts (supplementary S1.2).

### Chromatin Immunoprecipitation

Cells were grown to 80% confluence in T175 flasks and treated for 1 hour under one of the following conditions: basal (untreated), 1 or 10 µM cocaine or vehicle alone. Samples were processed following methods described by Murgatroyd et al.^31^ Immunoprecipitation was performed using anti-NRSF (H-290) (Santa Cruz Biotechnology), which recognizes amino acids 1–290 of human REST. PCR analysis of the immunoprecipitated chromatin samples was performed using primers targeting 2 predicted REST binding sites (BS) across the MIR137 locus (figure 1) and BDNF promoter 2 as a positive control for REST binding.^27,32^ Primer sequences are detailed in supplementary S1.2.

## Results

### Bioinformatic Analysis of the Human MIR137 Locus

MIR137 is located on chromosome 1p22 within the nonprotein coding RNA genes MIR137HG (MIR137 host gene/AK094607),^7^ AK311400,^33^ and AK309618. Within the primary (pri)-miRNA-137 sequence, there is a 15-bp VNTR, 6bp upstream of the precursor (pre)-miRNA-137 from which the functional mature miRNA-137 (miR-137) is processed^7^ (figure 1A). ChIP-seq data from ENCODE^34,35^ identified only RNA Polymerase II and the transcription factor REST binding over the MIR137 VNTR (position, chr1:98511662-98511917, hg19). ENCODE data also identified REST occupancy within the first intron of MIR137HG (chr1:98513800-98514144, hg19). The rVista portal^36^ also supported REST binding at the VNTR adjacent to pre-miR-137 (position, chr1:98511764-98511782, hg19) from the Transfac database.^37^ The genomic architecture surrounding the MIR137 gene, such as active histone marks and CpG islands identified from UCSC data, suggested a promoter in this region, and we hypothesized it could be important for the regulation of miR-137 expression and the expression of neighboring transcripts. We termed this putative internal promoter, Imir137 promoter.

### Imir137 Promoter Supports Luciferase Reporter Gene Expression in the SH-SY5Y Neuroblastoma Cell Line

**Fig. 2.**
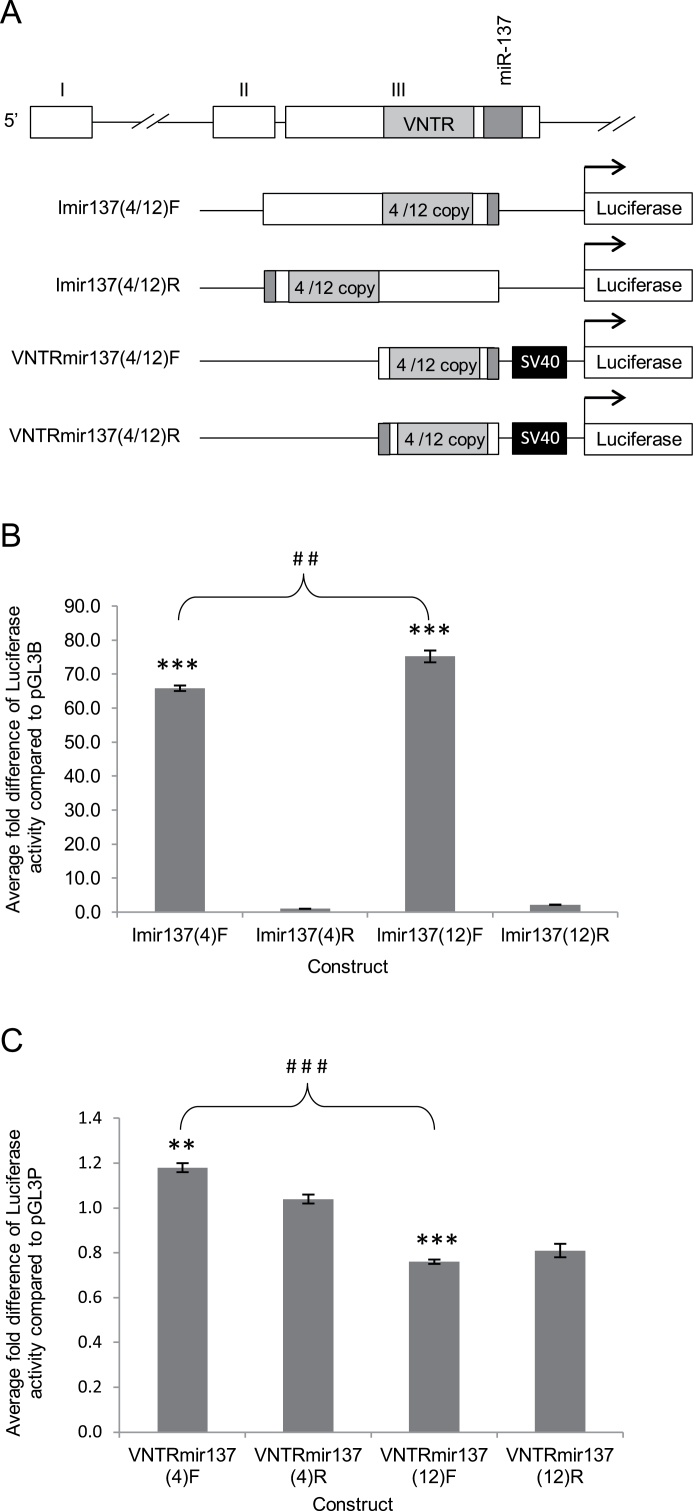
Validation of an internal promoter in the MIR137 gene. (A) Schematic representation of MIR137 constructs aligned to the MIR137 gene showing the 4-copy (Imir137(4)) and 12-copy (Imir137(12)) variants of the MIR137 VNTR ± the proximal flank region in the pGL3B (Basic) and pGL3P (Promoter) vectors, respectively, in forwards (F) and reverse (R) orientation. (B,C) Average fold change in luciferase activity supported by the MIR137 constructs over vector controls in SH-SY5Y cells. *N* = 4. *Significant changes in luciferase activity over backbone control. ^#^Significant changes in luciferase activity between experimental conditions. */^#^ *P* < .05, **/^##^ *P* < .01, ***/^###^ *P* < .001.

To assess the function of the putative Imir137 promoter, we cloned it into the pGL3-Basic (pGL3B) luciferase reporter gene vector. We tested 2 promoter variants, containing either a 4- or 12-copy VNTR, specifically we included nucleotides −361/−481 to +38 numbered from the first base of the pre-miR-137 sequence as +1 (figure 2A). One previous report demonstrated that the low (3-copy) and high (12-copy) VNTRs differentially affected miR-137 processing in vitro.^7^ We similarly compared their ability to modulate transcription. The difference in the 5′ end of these fragments reflected the two distinct VNTRs and were termed Imir137(4) and Imir137(12), respectively. We clearly demonstrated the ability of Imir137 to act as a promoter, observing a 65.0- or 75.0-fold increase in activation when containing either the 4- or 12-copy repeats, respectively, in the forward orientation (figure 2B, ***P < .001). Consistent with promoter function in this domain, the reverse orientation had no activity. A small but significant difference (##*P* < .01) was observed when the two alleles were compared with one another.

To determine whether any effect was contributed by the VNTR alone, we cloned 2 variants of this domain, either −86 or −206 to +38bp (reflecting the 4- and 12-copy repeats) into the pGL3-Promoter (pGL3P) vector, upstream of the minimal SV40 promoter in forward and reverse orientations (figure 2A). These were termed VNTRmir137(4)F or R and VNTRmir137(12)F or R, respectively, to indicate both copy number and orientation of fragment. The 4-copy variant supported a small increase in reporter activity, whereas the 12-copy variant repressed activity; this resulted in a significant difference in activity when the 2 VNTRs in the forward orientation were compared with one another (figure 2C; ###*P* < .001).

### REST Can Bind to the Imir137 Promoter Region and Modulate Its Activity in a Stimulus-Inducible and Allele-Dependent Manner

**Fig. 3.**
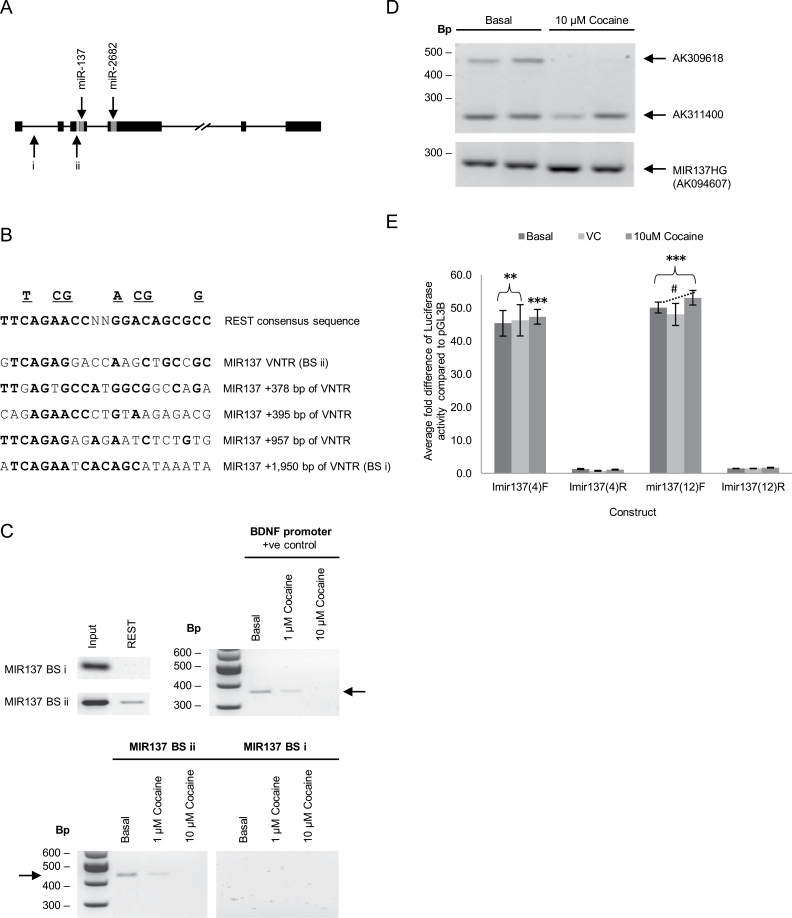
REST modulation of the MIR137 internal promoter in SH-SY5Y cells. (A) Predicted REST binding sites (BS) across MIR137 identified using *Transcription Factor ChIP-seq from ENCODE,* UCSC Genome Browser (http://genome.ucsc.edu/). (B) Canonical 21bp binding motif found within REST target genes and sequence homology with predicted BS within MIR137. (C) Chromatin immunoprecipitation (ChIP) assays showing REST binding at the putative BSii encompassing the internal MIR137 promoter (Imir137) VNTR under basal conditions and after 1 hour treatment with 1 and 10 µM cocaine. (D) Expression profiling of mRNAs expressed from the MIR137 locus after 1 hour treatment with 10 µM cocaine. (E) Relative levels of luciferase expression supported by the Imir137(4/12)F/R constructs in response to 1 hour treatment with 10 µM cocaine. *N* = 4. *Significant changes in luciferase activity over backbone control levels. ^#^Significant changes in luciferase activity between experimental conditions. */^#^ *P* < .05, **/^##^ *P* < .01, ***/^###^ *P* < .001.

There are 2 predicted REST BS at the MIR137 locus, which we term BSi and BSii (figures 3A and 3B). To validate REST occupancy, we performed chromatin immunoprecipitation (ChIP) assays in SH-SY5Y cells that are homozygous for the 4-copy repeat variant of the MIR137 promoter VNTR. The antibody recognizes the amino terminal of REST and therefore identifies all known isoforms of this protein. SH-SY5Y cells express both full-length REST and the sNRSF isoform. REST binding was observed over the Imir137 promoter (BSii) but not at the intronic site of MIR137HG (BSi) (figure 3C). To investigate a potential role for REST-mediated regulation of the MIR137 locus, we treated SH-SY5Y cells with cocaine, which is known to modulate many genes regulated by REST.^38^ After 1 hour of treatment with 1 µM cocaine, REST binding was reduced at the Imir137 promoter (BSii), with loss of binding observed after 10 µM cocaine treatment (figure 3C). As a control, we demonstrated that cocaine reduced REST binding at the previously characterized BDNF RE1/NRSE domain (figure 3C).^27,32^ The loss of binding to BSii was correlated with loss of AK309618 mRNA expression but not MIR137HG and AK311400 expression (figure 3D). Cocaine had no effect on the level of reporter gene expression supported by either Imir137(4) or Imir137(12), which might suggest other domains outside the proximal promoter are involved in the response of the endogenous gene (figure 3E).

**Fig. 4.**
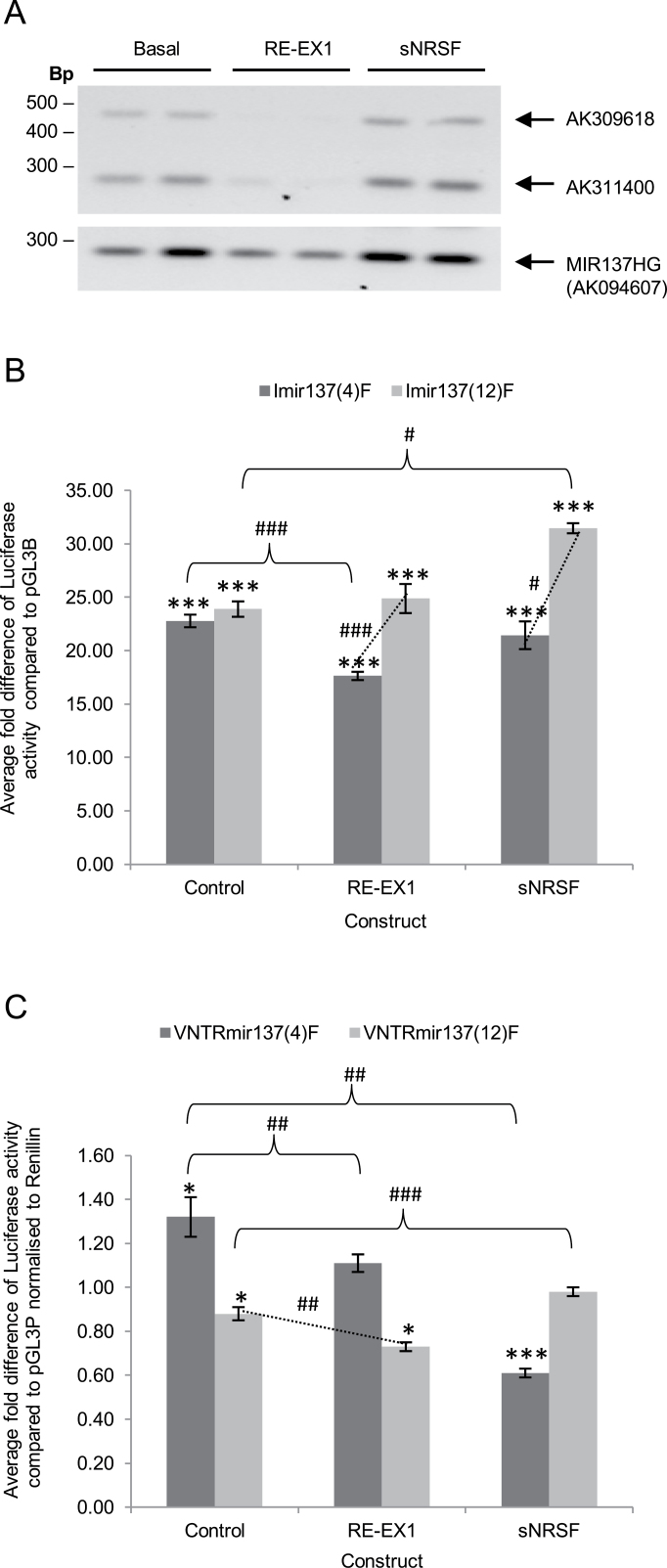
Differential regulation of the MIR137 locus after REST overexpression in SH-SY5Y cells. (A) Expression of AK311400, AK309618, and MIR137HG/AK094607 mRNA after overexpression of full-length human REST (RE-EX1) and sNRSF. Expected band sizes for AK311400, AK309618, and MIR137HG were 274, 451, and 291bp, respectively. (B,C) Average fold change in luciferase activity compared to pGL3B (B) and pGL3P (C) controls after transfection of MIR137 reporter gene constructs under basal conditions or in combination with RE-EX1 or sNRSF overexpression constructs. *N* = 4. *Significant changes in luciferase activity over backbone control. ^#^Significant changes in luciferase activity between experimental conditions. */^#^ *P* < .05, **/^##^ *P* < .01, ***/^###^ *P* < .001.

We analyzed the affect of overexpression of full-length REST (RE-EX1) and sNRSF on both endogenous MIR137 mRNA and reporter gene constructs containing the internal promoter in SH-SY5Y cells. Overexpression of RE-EX1 but not sNRSF resulted in down-regulation of both AK311400 and AK309618 mRNAs whose transcripts initiate from the Imir137 promoter region (figure 4A). This was not a metabolic or nonspecific affect on the cell transcriptome because MIR137HG expression was not affected (figure 4A). Co-transfection of the Imir137 promoter constructs demonstrated a differential response to either RE-EX1 or sNRSF, dependent on the genotype of the VNTR. RE-EX1 overexpression demonstrated a decrease in reporter gene activity on the Imir137(4) (*P < .05), whereas the Imir137(12) was not affected (figure 4B). Conversely, sNRSF had no affect on Imir137(4) but increased activity supported by Imir137(12). The difference between activity supported by Imir137(4) and Imir137(12) in response to RE-EX1 and the truncated isoform sNRSF was 1.4-fold (###*P* < .001) and 1.5-fold (#*P* < .05), respectively.

When the VNTR domain alone was investigated in conjunction with RE-EX1 overexpression, it was noted that a small and comparable reduction in activity (##*P* < .01) was seen for both VNTRmir137(4)F (1.2-fold) and VNTRmir137(12)F (1.4-fold) (figure 4C). However, the greatest difference observed was in response to sNRSF overexpression, which resulted in major repression of the 4-copy VNTR (2.2-fold). There is a potential REST BS within the MIR137 VNTR sequence domain (figure 1B).

### Allele Frequency of the MIR137 VNTR

To investigate VNTR heterogeneity, we determined both allele frequency within the HapMap CEU population and linkage to the previously identified GWAS SNP, rs1625579. Our analysis indicated that the VNTR was not in linkage disequilibrium (LD) with the GWAS SNP (supplementary figure S1); however, we identified a large number of variants (4–12 copies) of the VNTR within the CEU population, with a plurality of individuals (55%) homozygous for the 4-copy variant (Table 1). However, the higher copy-number variants of the VNTR were rare, thus giving us little power to address the role of the functionally distinct 12-copy variant found both in this communication and previously as a modulator of miR-137 processing in a cell line model,^7^ as a simple genetic correlate for risk to schizophrenia. Furthermore, we searched for LD using the CEU sample panel, integrating the VNTR genotypes using a down coding procedure whereby each allele is coded as a SNP depending on its dosage (0,1,2 becoming 11,12,22). None of the SNPs in high LD with the previous GWAS finding showed LD with *R* ^2^ > .05. These results are compatible with a VNTR mutating by both recombination and homoplasy.

**Table 1.** Genotype and Allele Frequency Data for the MIR137 Variable Number Tandem Repeat (VNTR) in the HapMap CEU Cohort

	Number of Counts	Frequency (%)
Genotype		
4 4	49	55.06
4 5	9	10.11
4 6	10	11.24
4 7	3	3.37
4 8	3	3.37
4 9	2	2.25
4 10	5	5.62
4 11	1	1.12
4 12	1	1.12
5 6	2	2.25
6 6	1	1.12
6 8	2	2.25
6 9	1	1.12
Total	89	
Allele		
4	132	74.16
5	11	6.18
6	17	9.55
7	3	1.69
8	5	2.81
9	3	1.69
10	5	2.81
11	1	0.56
12	1	0.56
Total	178	

*Note*: Numbers represent copy number of the repeat.

## Discussion

The genomic architecture of the MIR137 gene suggested the presence of a promoter in the region adjacent to the sequence of miR-137 itself and thus internal to the main precursor message. We termed this putative promoter region Imir137. The structure of this promoter was also of interest as it contained a VNTR domain 6bp upstream of the precursor sequence of miR-137. We confirmed the existence of this novel promoter by reporter gene analysis and validated the presence of 2 mRNAs that originate in this area of the gene locus, AK311400 and AK309618 (figures 2 and 3). We validated ENCODE data that the REST transcription factor bound at this promoter, figure 3. To address whether REST was involved in the differential regulation of mRNAs at the Imir137 promoter, we modulated the levels of REST with an expression construct. This resulted in a decrease in both transcripts originating at the Imir137 promoter but no affect was observed on the expression of the full-length MIR137HG. This was consistent with the binding observed in ChIP for REST at the internal promoter. We further determined that the genotype of the VNTR in the Imir137 promoter region could mediate differential reporter gene expression. This latter observation was in the presence of overexpressed full-length REST or a truncated isoform, sNRSF. The proximal Imir137 promoter and the VNTR were found not to be in disequilibrium with the GWAS SNP for schizophrenia (supplementary figure S1), and therefore, the regulation of this promoter supplies a distinct pathway for modulation of miR-137 levels.

Modulation of epigenetic pathways by REST in association with the SWI/SNF chromatin remodeling complex^21^ has been implicated in schizophrenia. A similar model involving REST could operate at the MIR137 locus, in particular at the Imir137 promoter; furthermore, such a mechanism could be modified by any genetic variants embedded at the gene locus, thus further modulating miR-137 levels. Regulation by REST is complicated by the distinct functions that have been assigned to various isoforms of this gene. The proteins corresponding to these isoforms have not been examined as extensively as the full-length protein. The isoform sNRSF used in our current study results from the insertion of a small exon (N) between the accepted third and fourth exons; the inclusion of this exon results in a frame-shift and, with it, the introduction of a stop codon at the start of exon 4 resulting in a truncated protein.^26^ This is similar to the REST4 isoform in rodents.^27,28^ Overexpression of sNRSF resulted in data that were distinct from the action of full-length REST on both the endogenous MIR137 gene and several of the reporter gene constructs. Mechanistically, the different REST isoforms have been suggested to have distinct regulatory functions, for example, full-length REST represses the human synapsin I promoter, whereas the truncated REST variant does not.^39^ Alternatively, the truncated isoform has been proposed to act as an antagonist of REST-mediated gene repression, for example, REST repression of both the BDNF promoter and the cholinergic gene locus has been shown to be alleviated by the truncated isoform.^40,41^ Our data on the action of REST or the truncated variant on the regulation of the endogenous MIR137 gene or the reporter gene constructs are consistent with this differential action of these distinct REST isoforms. However, SH-SY5Y cells endogenously express both isoforms and overexpression modulates the ratio of REST/sNRSF in the cell. We predict that in schizophrenia, environmental challenges and stress might result in alterations in the ratios of REST isoforms that could ultimately result in an altered pattern of gene expression. Significantly, the data from reporter gene constructs indicated that the genotype of the VNTR could modulate the response to the change in REST levels. This would suggest that a gene × environment (G × E) factor is driving the modulation of expression over the locus.

We identified 3 mRNAs produced over the MIR137 locus in SH-SY5Y cells: MIR137HG (AK094607), AK311400, and AK309618, figures 2 and 4. The differential regulation of transcripts from the major MIR137 promoter and Imir137 would be expected to not only vary the levels of miR-137 but also the ratio of miR-137 to a second microRNA at this locus, miR-2682 (figure 1). The function or significance of miR-2682 has not been addressed in previous communications. MiR-2682 is not present in the AK311400 transcript. Similarly, the different transcripts expressed from this region may be posttranscriptionally processed, leading to different levels of either microRNA, which could result in central nervous system (CNS) dysfunction. In vivo, the modulation of REST levels that modulate the transcripts expressed could be directed by psychological stressors or other trauma; however, the action of the drug used in this communication validates the plasticity in expression at this locus. Cocaine, a known modulator of REST expression, resulted in the loss of expression of AK309618—one of the 2 transcripts predicted to originate at the internal promoter region. There was no affect on the expression of AK311400, which would originate from a similar position, and the full-length MIR137HG (AK094607) transcript (figure 3). We cannot determine here whether REST levels modulate the differential expression of transcripts directed by the Imir137 promoter in response to cocaine; only that this drug induces a specific set of transcriptional responses acting at the internal promoter, of which REST is one. Nevertheless, the data are consistent with a central role for REST in modulating the activity of the Imir137 promoter.

Identification of the VNTR was of interest in part because the copy number of this type of repetitive domain within the promoter of other genes we have analyzed, such as MAOA, SLC6A3 or SLC6A4, both correlates with susceptibility to a CNS disorder and supports differential reporter gene expression.^14–17,42–46^ We demonstrated that reporter gene function of the Imir137 promoter was modulated by the genotype of the VNTR; we tested the 4- and 12-copy variants based on a previous study that addressed a 3- and 12-copy variant in a cell line model of cancer for its ability to modulate the processing of the mature miR-137.^7^ We did not test each individual repeat in vitro because although we could perform reporter gene analysis, it would be difficult to correlate with ChIP data on the full set of variants. However, we demonstrated that the VNTR was not in disequilibrium with the GWAS SNP (supplementary figure S1). Using the HapMap CEU population, we demonstrated a large number of variants (4–12 copies) of the VNTR, although the majority of individuals (55%) were homozygous for the 4-copy variant (Table 1). The higher VNTR copy-number variants are rare within the population, thus giving us little power to address the role of the functionally distinct 12-copy variant as a simple genetic correlate for risk to schizophrenia. Furthermore, it might also suggest that the larger copy variants are selected against; further work will be required to resolve the role of the VNTR, if any, in schizophrenia risk.

In summary, the internal Imir137 promoter described in this communication can be regulated in an allele-specific and stimulus-inducible manner in part by the transcription factor REST. REST could modulate epigenetic parameters in response to environmental factors, such as stress, in the medium to long term in addition to the immediate changes observed in our cell line model. This is consistent with a G × E mechanism regulating miR-137, and potentially miR-2682, levels in the cell, with alterations in the concentration of these miRNAs resulting in differential repression of their gene targets in response to environmental cues. This differential expression directed by REST could be further affected by VNTR genotype. The resulting levels of these miRNAs could play a significant role in CNS dysfunction, including schizophrenia. This mechanism to modulate miR-137 levels is distinct from the mechanism associated with the intronic GWAS SNP, which we found not to be in disequilibrium with this internal promoter.

## Supplementary Material

Supplementary material is available at http://schizophreniabulletin.oxfordjournals.org.

## Funding

This work was supported by the Biotechnology and Biological Sciences Research Council (BB/F016905/1 to A.W.; V.J.B.; J.P.Q.).

## Supplementary Material

Supplementary Data
