# Supplementary material for: Characterization of a REST-Regulated Internal Promoter in the Schizophrenia Genome-Wide Associated Gene MIR137
Source: Schizophr Bull. 2014 Aug 25;41(3):698–707. doi: 10.1093/schbul/sbu117 (PMC4393679; doi:10.1093/schbul/sbu117)
Supplement: Supplementary Data [file supp_41_3_698__index.html]

Characterization of a REST-Regulated Internal Promoter in the Schizophrenia Genome-Wide Associated Gene MIR137 — Characterization of a REST-Regulated Internal Promoter in the Schizophrenia Genome-Wide Associated Gene MIR137 — Supplementary Data 

# Characterization of a REST-Regulated Internal Promoter in the Schizophrenia Genome-Wide Associated Gene MIR137

## Supplementary Data

Data files

**Files in this Data Supplement:**

- Supplementary Data - Supplementary Data
