# Supplementary material for: Characterization of a REST-Regulated Internal Promoter in the Schizophrenia Genome-Wide Associated Gene MIR137
Source: Schizophr Bull. 2014 Aug 25;41(3):698–707. doi: 10.1093/schbul/sbu117 (PMC4393679; doi:10.1093/schbul/sbu117)
Supplement: Supplementary Data [file supp_sbu117_Supplemental_Material_S1.pdf]

Supplemental Information

S1. Methods

S1.1. Plasmid Construction

MIR137 fragments were amplified from human genomic DNA using Phusion DNA polymerase (NEB) and the following oligonucleotide primers from Eurofins MWG Operon: rsMIR137_F: 5'–ATAC(CTCGAG/ACGCGT)ACCCAAGAATACCCGTCA–3' and rsMIR137_R1: 5'–ATAC(ACGCGT/CTCGAG)TGGGAGAGCACCAGGTAAA–3', targeting the VNTR plus flanking sequence for cloning into the pGL3-Basic (pGL3B) luciferase reporter vector (Promega) and the alternative reverse primer rsMIR137_R2: 5'–ATAC(ACGCGT/CTCGAG)AGCAGCAAGAGTTCTGGT-3', targeting the VNTR alone for cloning into the pGL3-Promoter (pGL3P) vector (Promega) containing an SV40 promoter. Sequences in brackets indicate the XhoI/MluI restriction sites (CTCGAG/ACGCGT) for direct cloning in the forward and reverse orientations. Constructs were made containing a 4-copy and 12-copy repeat of the VNTR in both the forward and reverse orientation and named Imir137(4)F/R and Imir137(12)F/R, respectively, for the pGL3B constructs used to address promoter function and VNTRmir137(4)F/R and VNTRmir137(12)F/R for the pGL3P constructs used to assess the functional activity of the different alleles of the VNTR in both orientations. DNA sequencing confirmed the correct sequence for each construct.

S1.2. PCR Primers

For gene expression profiling of the MIR137 host genes: AK311400_F: 5'–ACTCTCTTCGGTGACGGGTA–3' and AK311400_R: 5'–TCCACTCTGGGTCATCCTTC–3', targeting both the AK311400 and AK309618 mRNAs, and MIR137HG_F: 5'-CAGAGGAAAGCACTGGGAGA-3' and MIR137HG_R: 5'-CACCCAAGAATACCCGTCAC-3', targeting the mRNA sequence for MIR137HG. For chromatin immunoprecipitation (ChIP): MIR137_BSi_F: 5'-ACCTACCCAATGTTCCACCA-3' and MIR137_BSi_R: 5'-CGACAGCTTAAGGAGGCTTG-3', targeting the REST binding site (BS) i within intron 1 of MIR137HG; MIR137_BSii_F: 5'-CACCCAAGAATACCCGTCAC-3' and MIR137_BSii_R: 5'-TGGGAGAGCACCAGGTAAAC-3', targeting REST BS ii within the internal promoter VNTR and BDNF_F: 5'-

GAGATTTTTAAGCCTTTTCCTC-3’ and BDNF_R: 5’-CTTGCCAAGAGTCTATTCC-3’ targeting the well characterised REST BS within promoter 2 of BDNF.

S1.3. Genotyping and Linkage disequilibrium (LD) Analysis

The MIR137 VNTR was amplified using 5ng genomic DNA from individuals from the CEPH HapMap cohort and GoTaq® Flexi DNA polymerase (Promega) using PCR primers: MIR137_F: 5’-CACCCAAGAATACCCGTCAC-3’ and MIR137_R: 5’-TGGGAGAGCACCAGGTAAAC-3’. PCR products were analysed by gel electrophoresis. Expected fragment sizes ranged between 400-520 bp, ±15 bp. Duplicate samples were tested and negative controls included. Genotype data was uploaded into Haploview version 4.2 along with individually matched genotype data for common SNPs spanning the genomic locus (chromosome 1: 98,075,522-98,711,836), downloaded from the HapMap Genome Browser, release #28 (http://hapmap.ncbi.nlm.nih.gov/index.html.en). R^2 plots were generated using the Linkage Format feature (Hardy-Weinberg p-value cut-off, 0.001; minimum genotype cut-off, 75%; maximum number of Mendel errors, 1; minimum minor allele frequency, 0.01) and pair-wise tagging analysis performed (r^2 threshold, 0.8) to determine LD between the GWAS SNP rs1625579 and the VNTR.
